# Increased of exhaled breath condensate neutrophil chemotaxis in acute exacerbation of COPD

**DOI:** 10.1186/s12931-014-0115-0

**Published:** 2014-09-28

**Authors:** Jean Louis Corhay, Catherine Moermans, Monique Henket, Delphine Nguyen Dang, Bernard Duysinx, Renaud Louis

**Affiliations:** Department of Pneumology, CHU, Sart-Tilman B-35, Liège, 4000 Belgium; GIGAi3 Research group, University of Liege, Liege, 4000 Belgium; Department of Pneumology, CHU Sart-Tilman B-35, GIGAi3 Research group, University of Liege, Liège, 4000 Belgium

**Keywords:** Airway inflammation, Chronic obstructive pulmonary disease, Exacerbation, Exhaled breath condensate, Growth related oncogen-α, Leukotriene B4, Neutrophil chemotactic activity

## Abstract

**Background:**

Neutrophils have been involved in the pathogenesis of chronic obstructive pulmonary disease (COPD). Underlying mechanisms of neutrophil accumulation in the airways of stable and exacerbated COPD patients are poorly understood. The aim of this study was to assess exhaled breath condensate (EBC) neutrophil chemotactic activity, the level of two chemoattractants for neutrophils (GRO-α and LTB4) during the course of an acute exacerbation of COPD (AECOPD).

**Methods:**

50 ex smoking COPD patients (33 with acute exacerbation and 17 in stable disease) and 20 matched ex smoking healthy controls were compared. EBC was collected by using a commercially available condenser (EcoScreen®). EBC neutrophil chemotactic activity (NCA) was assessed by using Boyden microchambers. Chemotactic index (CI) was used to evaluate cell migration. LTB_4_ and GROα levels were measured by a specific enzyme immunoassay in EBC.

**Results:**

Stable COPD and outpatients with AECOPD, but not hospitalized with AECOPD, had raised EBC NCA compared to healthy subjects (p < 0.05 and p < 0.01 respectively). In outpatients with AECOPD EBC NCA significantly decreased 6 weeks after the exacerbation. Overall EBC NCA was weakly correlated with sputum neutrophil counts (r = 0.26, p < 0.05).

EBC LTB4 levels were increased in all groups of COPD compared to healthy subjects while GRO-α was only raised in patients with AECOPD. Furthermore, EBC LTB_4_ and GRO-α significantly decreased after recovery of the acute exacerbation. Increasing concentrations (0.1 to 10 μg/mL) of anti- human GRO-α monoclonal antibody had no effect on EBC neutrophil chemotactic activity of 10 exacerbated COPD patients.

**Conclusions:**

EBC NCA rose during acute exacerbation of COPD in ambulatory patients and decreased at recovery. While LTB4 seems to play a role both in stable and in exacerbated phase of the disease, the role of GRO-α as a chemotactic factor during AECOPD is not clearly established and needs further investigation.

## Background

Chronic obstructive pulmonary disease (COPD) is characterized by airway remodelling and an inflammatory cell infiltrate in which neutrophils play a key role [1]. The extent of neutrophilic infiltration in both the airway lumen and tissues is correlated with COPD severity [2–4], is associated with bronchial colonisation [5] and forced expiratory volume in one second (FEV_1_) decline in stable COPD patients [5,6] and is amplified during exacerbation [7,8]. However neutrophil accumulation mechanisms in the airways are poorly understood. They could involve increased neutrophil influx with or without increased survival of these cells.

There has been growing interest in the use of exhaled breath condensate (EBC) as a non-invasive method for assessing inflammation in many lung diseases such as COPD [9–12]. This technique has the advantage of being particularly well tolerated by patients with an acute exacerbation of COPD (AECOPD) [13,14]. Many biomarkers and components can be measured or detected in EBC, which are derived from aerosolised particles from the whole bronchial tree (proximal and distal airways) [9]. Some of them are chemoattractants for neutrophils (Leukotriene B4 (LTB_4_), Growth related oncogen-α (GRO-α), Interleukin-8 (IL-8)). However the detection of IL-8 in EBC remains problematic because its level is close to the lower limit of detection in currently available immunoassays [14–16].

On the other hand the biological activity of EBC has been poorly investigated so far. In a previous study [17], we found that active smoking and COPD were associated with raised EBC neutrophil chemotactic activity (NCA), and demonstrated the contribution of IL-8 and LTB_4_ to this activity in stable disease [18].

COPD patients are characterized by periods of exacerbation of their illness (AECOPD) and recurrent AECOPD contribute to the decline in lung function, decrease of quality of life and increased morbidity and mortality [19]. Most exacerbations of COPD are due to an airway infection [19]. During bacterial AECOPD, the neutrophils and neutrophil products (myeloperoxydase and elastase) as well as the neutrophil chemotactic attractants (IL-8 and LTB4) were found to temporarily increase in sputum and EBC before returning to lower levels after treatment [13,20–23].

The aim of this study was to assess the EBC NCA, GRO-α and LTB4 during the course of an acute exacerbation of COPD.

## Methods

### Study design and subject characteristics

**Figure 1:**
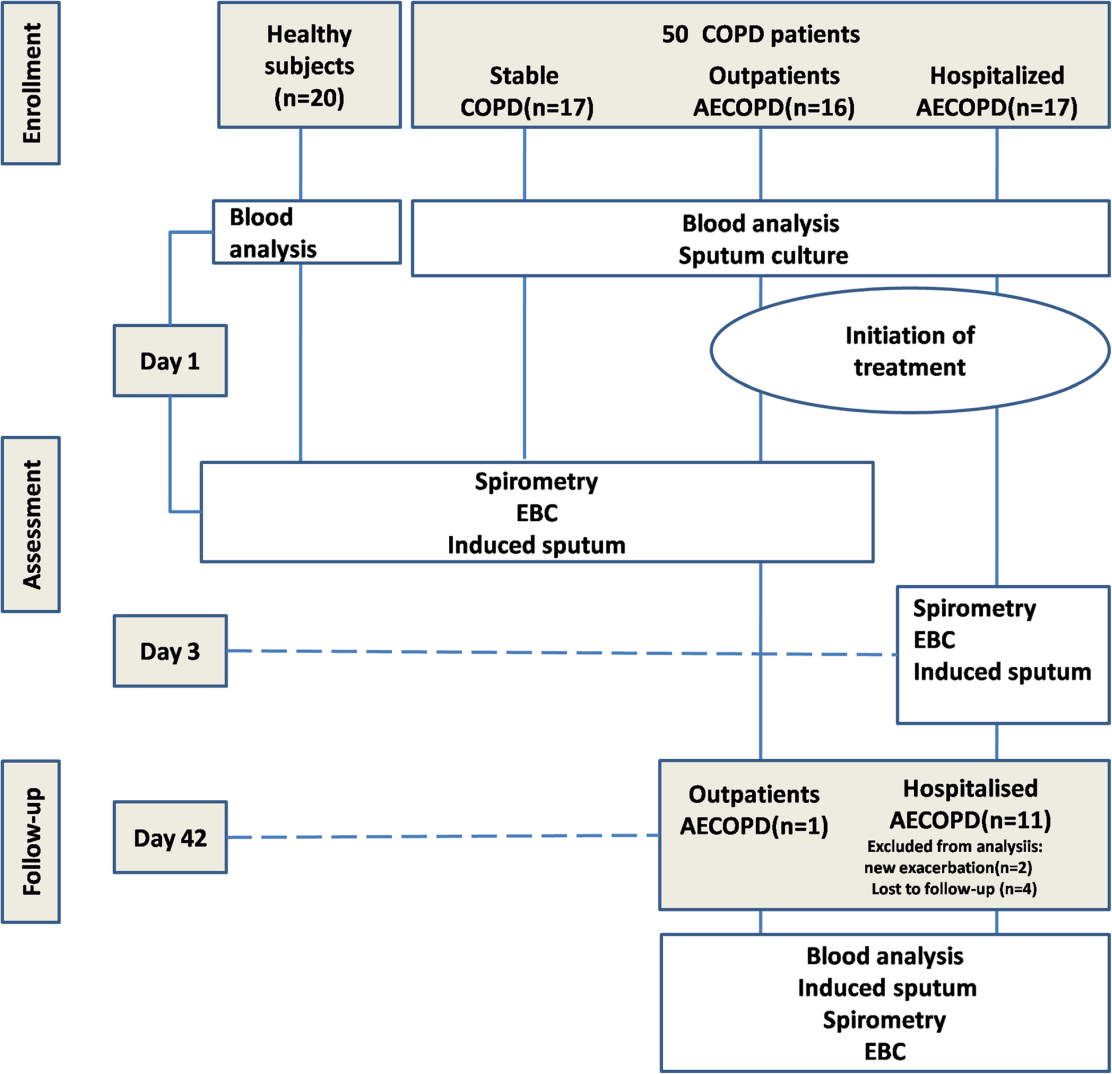
**Flow chart showing the study design.**

We prospectively recruited 50 ex-smoking moderate to very severe COPD patients (17 with AECOPD leading to hospitalisation, 16 outpatients with AECOPD and 17 in stable disease) and 20 ex-smoking healthy controls. The subgroup of COPD patients with AECOPD was longitudinally followed (Figure 1). The demographic, functional and treatment characteristics of the subjects are given in Table 1. All the COPD patients fulfilled the criteria proposed by the Global Initiative for Chronic Obstructive Lung Disease (GOLD) guidelines [19].

**Table 1:** **Demographic, functional and therapeutic characteristics**

	**Healthy controls**	**Stable COPD patients**	**Exacerbated COPD patients**	
			**Outpatients**	**Hospitalized**
No of subjects	20	17	16	17
Age (yrs)	57.1 ± 8.7	59.8 ± 7.4	61.8 ± 6.4	63.8 ± 8.4
Male/female number	8/3	13/4	13/3	13/4
BMI (kg/m^2^)	24.2 ± 3.3	25.8 ± 3.4	26.1 ± 4.8	26.7 ± 7.1
Number of pack years	25.2 ± 13.8	41.3 ± 18.9 †	41.4 ± 11.8 †	43.3 ± 14.3 ††
FEV1,% predicted value	99.6 ± 14.8	52.5 ± 18.0 †††	48.1 ± 11.6 †††	37.0 ± 14.1 †††*
FEV1/FVC%	77.5 ± 4.7	50.7 ± 11.7 †††	47.0 ± 13.3 †††	46.2 ± 9.5 †††
GOLD stages No				
GOLD 2		11	10	4*#
GOLD 3-4		6	6	13*#
Inhaled corticosteroid				
Number		13	15	14
Mean daily dose of ICSμg^¶^		1235.3 ± 877.1	1421.9 ± 553.2	1588.2 ± 678.6
Long-acting B2 agonist, number		13	15	14
Long-acting Anticholinergic, number		15	14	16
Oral steroids^¶¶^, number		0	0	17

Data are expressed as means ± SD.

^¶^Expressed as beclomethasone equivalent (In converting corticosteroid drugs to a beclomethasone equivalent, we assumed that 1 mg of beclomethasone was equivalent to 0.8 mg of budesonide, 0.5 mg of fluticasone).

^¶¶^Taken at the moment of sampling.

† p < 0.05, †† p <0.01, ††† p < 0.001 vs controls. *p < 0.05 vs stable COPD, #p < 0.05 vs outpatients with exacerbation of COPD.

COPD: chronic obstructive pulmonary disease; BMI: body mass index; FEV_1_: forced expiratory volume in one second; FVC: forced vital capacity.

Outpatients with AECOPD were recruited from the consultations and hospitalized patients when admitted into the ward (Department of Pneumology, CHU of Liège). If patients were already treated with antibiotics and/or systemic steroids at the admission in the hospital, they were not being included in the study. AECOPD is defined as an event in the natural course of the disease characterized by a change in the patient’s baseline dyspnoea, cough and/or sputum that is beyond normal day-to-day variations, is acute in onset, and may warrant a change in regular medication [24,25]. Sputum was defined as “purulent” when patients reported a change in the color of spontaneously expectorated samples over the past 72 h from uncolored to yellow-green [26]. Exacerbations were graded as moderate when requiring treatment with systemic corticosteroids and/or antibiotics and as severe if hospitalization or a visit to the emergency department was required. They were treated according to good clinical practice, outpatients (moderate AECOPD) received broad spectrum antibiotics and/or systemic steroids according to COPD severity and to Anthonisen criteria [27]. All hospitalized patients received systemic corticosteroids and antibiotics.

All sample collections and the measurements (EBC, sputum culture, spirometry and induced sputum) were performed during an exacerbation, before starting treatment for outpatients, and 72 h after starting treatment for hospitalized patients, and then repeated 6 weeks later when patients were in a stable condition.

Healthy subjects and stable COPD attended the laboratory for one single visit. COPD patients were all in stable condition at the time of EBC collection, blood sample collection and sputum induction, and no patient were studied within 12 weeks of any exacerbations requiring change in maintenance treatment or oral steroid and antibiotic prescription. Twenty ex-smoking subjects, with normal pulmonary function, were also studied as controls. They had not suffered from asthma or chronic bronchitis.

Subjects were considered as ex-smokers if they had stopped smoking for at least six months prior to the study. Ex-smoking status was checked by measuring urinary cotinine levels.

The protocol was approved by the ethics committee “Hospitalo-Facultaire Universitaire” of Liège (IRB: 2005/181), and subjects gave written informed consent for their participation.

### Exhaled breath condensate collection

Exhaled breath condensate (EBC) was collected by using a commercially available condenser (EcoScreen®, Erich Jaeger Viasys GmbH, Hoechberg, Germany), according to ATS/ERS recommendations [9], which yielded nongaseous components of the expiratory air. Subjects breathed through a mouthpiece connected to the condenser. They were asked to breathe at a normal frequency and tidal volume, wearing a nose clip, for a period of 10 min. Approximately 1.5 - 2 ml of EBC were collected and immediately stored (in 250 μL aliquots) at 80°C, within 5 min after collection, until analysis. All samples were tested for salivary contamination by determination of α-amylase concentration (enzymatic colorimetric assay with a detection limit of 3U/mL). The collection of EBC systematically preceded the sputum induction procedure.

### Neutrophil isolation

Neutrophils were isolated using a two step procedure from 40 ml of peripheral venous blood of the same non-atopic and non smoking healthy volunteer. The whole blood was treated with Geloplasma® (Institut Merieux Belgium) 9/1, for 45 minutes to sediment the red blood cells. The leukocytes-rich liquid phase was diluted three times with phosphate buffer saline (PBS) and was centrifuged through a Percoll® separating solution (Biochrom- AG- Germany) gradient density 1.077 (centrifugation at 400 g for 30 minutes at 22°C) to separate the mononuclear leukocytes from the granulocyte fraction (>95% neutrophils). Neutrophils were then washed twice in PBS and resuspended in Hank’s balanced salt solution (HBSS) with Ca^2 +^ and Mg^2+^ (pH 7.4) and 0.2% bovine serum albumine (BSA) at 1.10^6^ cells.mL^−1^.

### EBC Neutrophil chemotaxis

In this study, EBC neutrophil chemotactic activity were assessed by using Boyden microchambers as previously described [17,18]. Aliquots of 28 μL of EBC were placed in the lower chambers and 50 μL of cells suspension (10^6^ cells.mL^−1^) were placed in the upper chambers. The two chambers were separated by a polycarbonate polyvinylpyrrolidone (PVP) - free filter 0.8 μm Nuclepore® (Whatman, USA). Experiments were performed in triplicate in a 48-well microchemotaxis Boyden chamber incubated in 5% C0_2_ at 37°C for 60 min. The negative controls consisted of a solution of HBSS with Ca^2 +^ and Mg^2+^ (pH 7.4). Chambers were dismantled and nonmigrated cells were scraped from the upper surface of the filters. The filters were then immersed in methanol and stained with Diff-Quick. Migrated cells adherent to the lower surface were counted in 10 fields in each well at a magnification X 600. The results were expressed as the number of neutrophils having migrated/10 high power field. Chemotactic index (CI) was calculated by the ratio of cell migration with the EBC/cell migration with Hank’s Balanced Salt Solution (as control). A value of 1.0 representing no chemotactic activity. The optimal concentration of EBC that was used in chemotaxis assays was determined previously and was 1/1 of the original sample [17,18]. The reproducibility of repeat EBC neutrophil chemotactic activity measurements was assessed. The intraclass correlation coefficient and the Spearman correlation coefficient were 0.67 and 0.74 (p < 0.01) respectively [17].

### Sputum induction and processing

Sputum induction and processing were performed according to a technique used in routine in our service [28], and a differential leukocyte count was obtained using a cytospin stained with May-Grünwald Giemsa on 500 non squamous cells. A bacteriological analysis of induced sputum was also carried out for exacerbated COPD patients.

### Peripheral blood puncture and assessment of systemic inflammation

Venous blood was collected in Vacutainer tubes from an antecubital site immediately when controls and patients were included in the study. Blood cell values included hemoglobin, platelet count, WBC count, and the differential leukocyte count, fibrinogen and C-reactive protein (CRP) levels were determined according to the routine of the hospital.

### GRO-α and LTB_4_ measurements in EBC

LTB_4_ and GRO- *α* were measured by a specific enzyme immunoassay with a commercial kits (LTB_4_ : Cayman Chemical Company, Ann Arbor, Michigan, USA; GRO-α: R&D Systems Europe, Abingdon, UK) according to instructions provided by the manufacturer. Immunoassay detection limits were 13 pg/mL and 15.6 pg/mL for LTB_4_ and GRO-α respectively. The intra-assay and inter-assay variabilities of LTB_4_ and GRO-α were less than 10%.

### Determination of the contribution of GRO-α to EBC neutrophil chemotaxis

First, solutions of GRO-α at increasing concentrations (0.0126 to 126 nM) were added in the lower part of Boyden microchambers, while suspensions of neutrophils were added in the upper part to study neutrophil CI. Maximal stimulating concentration was 12.6 nM, the NCI was 4.5 ± 1.3 and significantly different from 1 (p < 0.05).

We also validated our anti-human GRO-α monoclonal antibody (Human CXCL1/GRO-α antibody (R&D Systems, Abingdon, UK)) by looking for the maximal inhibiting concentration on NCA induced by GRO-α at 12.6 nM. The maximal inhibiting concentration was 0.1 μg/ml (−48.2%). Thereafter, serial concentrations (0.1 μg/ml to 10 μg/ml) of anti-human GRO-α monoclonal antibody were added to EBC for 1 h at room temperature before assessing EBC chemotactic activities.

### Statistical analysis

Demographic and functional data were expressed as mean ± standard deviation (SD). EBC LTB_4_ and GRO-α levels, induced sputum cellularity and EBC neutrophil CI were expressed as median and interquartile range (IQR). When the data showed normal distribution, they were compared with a one-way ANOVA, followed by Tukey-Kramer’s post-hoc testing. When the data did not show a normal distribution, they were compared with the Kruskal-Wallis test followed by Dunn’s post-hoc testing. Comparison between the stable phase and the exacerbation phase were performed with a paired t-test or Wilcoxon test according to the normality of the distribution. CI for each group of samples was evaluated by one-sample “t” test versus a hypothetical mean of 1.0 (representing zero net chemotactic activity). Levels of measured mediators below the detection limit of the ELISA kits were arbitrarily assumed to be half of the detection limit value for statistical analysis. Correlations between variables were performed using Spearman’s rank correlation test. A p < 0.05 was considered as significant.

## Results

### Demographic, functional and microbiologic characteristics of subjects

Healthy ex-smokers, as well as ex-smoking COPD patients with stable disease or exacerbation were well matched for age, gender and BMI (Table 1). As expected, COPD patients exhibited a poorer lung function and a more important cumulative tobacco smoke exposure compared to healthy ex-smokers. Moreover, hospitalized patients were more severely limited as shown by a higher proportion of GOLD stage 3–4 (13/17 patients) compared to the two other groups of COPD (6/17 for stable and 6/16 for outpatients with AECOPD, both comparisons with p < 0.05).

Among the 17 patients hospitalized for AECOPD only 11 were analyzed at 6 weeks for the recovery: 2 patients had another exacerbation before the control visit and were excluded from the study and 4 did not come at the control visit. By contrast, all outpatients with AECOPD attended their control visit at 6 weeks.

Among outpatients with AECOPD, 8 on 16 had a positive sputum bacterial culture (4 streptococcus pneumoniae, 1 Haemophilus influenzae, 2 Pseudomonas aeruginosa and 1 Moraxella catarrhalis), while the culture was positive in 9 on 17 hospitalized AECOPD (2 streptococcus pneumoniae, 3 Haemophilus influenzae, 1 Moraxella catarrhalis, 1 Escherichia coli and 2 Pseudomonas aeruginosa).

### Airway and blood neutrophilic inflammation in COPD

**Figure 2:**
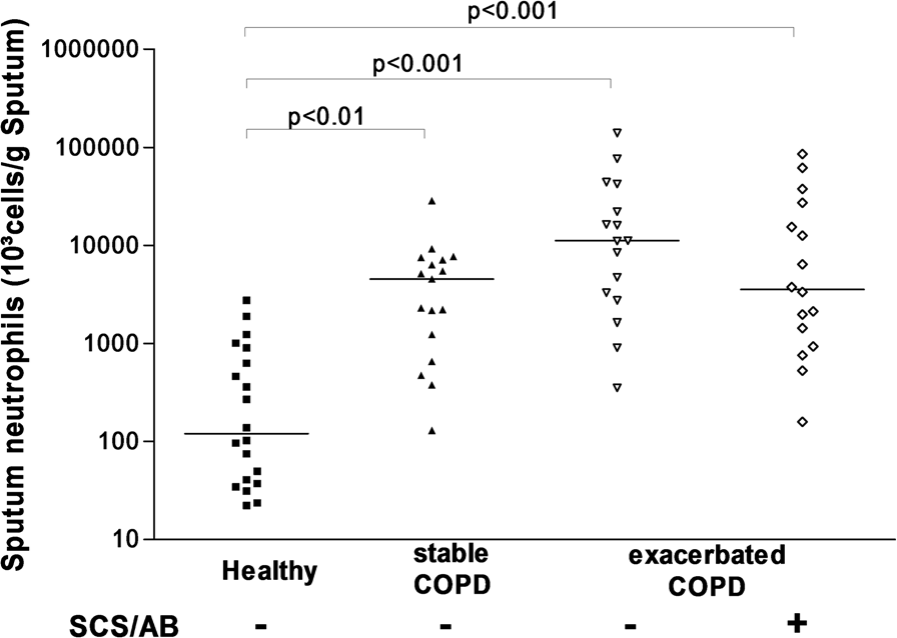
**Sputum neutrophil counts from stable and exacerbated COPD patients and healthy ex-smokers subjects.** Horizontal bars represent the median. SCS-AB: treatment with antibiotics and systemic corticosteroids before EBC collection.

**Figure 3:**
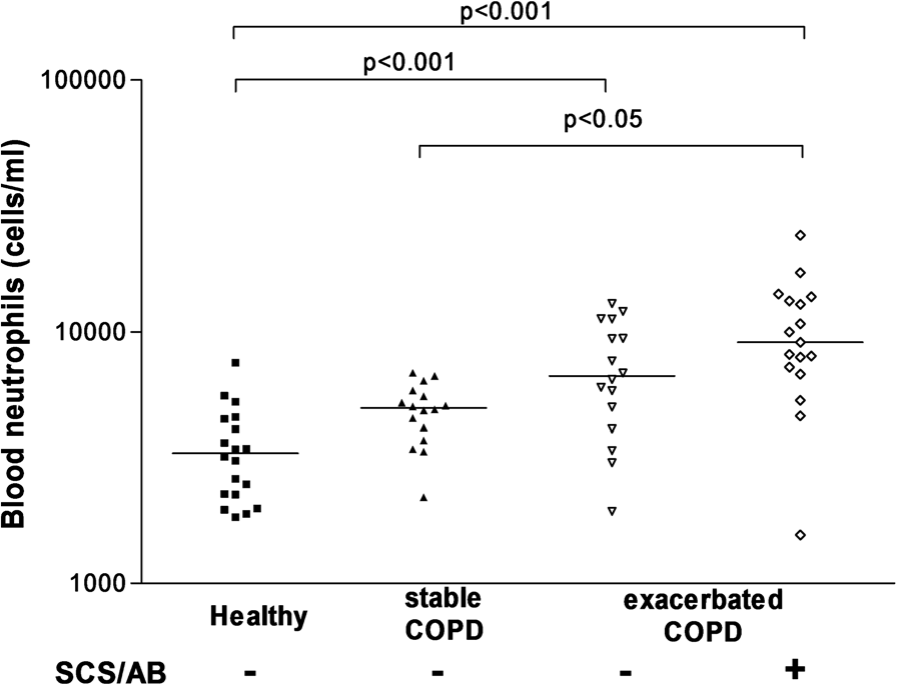
**Blood neutrophil counts from stable and exacerbated COPD patients and healthy ex-smokers subjects.** Horizontal bars represent the median. SCS-AB: treatment with antibiotics and systemic corticosteroids before EBC collection.

**Figure 4:**
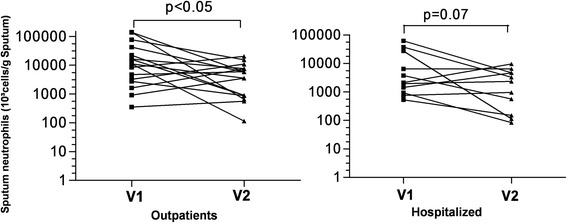
**Sputum neutrophil counts in acute phase (V1) and stable phase (V2) of outpatients and hospitalized patients with acute exacerbation of COPD.**

COPD was clearly characterized by an intense airway neutrophilic inflammation compared to healthy subjects (Table 2, Figure 2). Neutrophilic inflammation was further increased during exacerbation but this did not reach statistical significance. At blood level, exacerbated COPD displayed raised total leukocyte counts and a raised proportion of neutrophils (Figure 3). The levels of fibrinogen and CRP were also higher in blood of AECOPD patients compared to stable COPD patients and healthy subjects (Table 2). There was clear clinical improvement in patients 6 weeks after exacerbation as reflected by better spirometric indices and reduction in neutrophilic inflammation (Table 3 and Figure 4). We found a fall in mean blood neutrophils count of 4669/mm^3^ (95% Confidence Interval: 915–8424) and 2221/mm^3^ (95% Confidence Interval: 537–3906), and a fall in mean CRP of 24.05 mg/L (95% Confidence Interval: 1.36 - 46.73) and 43.46 mg/L (95% Confidence Interval: 13.76-72.97) respectively for hospitalized and outpatients with AECOPD.

**Table 2:** **Characteristic of blood and Induced Sputum samples, and EBC chemokines**

	**Healthy controls**	**Stable COPD patients**	**Exacerbated COPD patients**	
			**Outpatients**	**Hospitalized**
No of subjects	20	17	16	17
**Blood**				
Leukocytes (per mL)	5530 (3060)	7825 1420)	8825 (5040)*	12660 (5430)**†
Neutrophils (per mL)	3295 (1940)	4985 (1560)	6670 (5093)**	9090 (6030)**†
% neutrophils	60.1 (7.8)	59.2 (7.6)	76.0 (8.4)**††	84 (15.2)**†††
% lymphocytes	30.9 (7.3)	28.9 (8.6)	17.0 (10.1)*	10.8 (8.3)**†††
CRP (mg/L)	0.9 (2.1)	3.9 (5.9)	21.4 (83.5)**†	9.2 (49.0)**†
Fibrinogen (g/L)	2.6 (0.6)	3.3 (1.4)	4.9 (2.4)***†††	5.1 (1.7)***†
**Induced sputum**				
Squamous epithelial cells (%)	24.0 (25.0)	10.0 (10.0)	0.0 (2.0)**	6.0 (21.0)
Total non squamous cell count (x10^6^ cells/g sputum)	0.3 (1.2)	3.7 (6.4)*	13.1 (25.7)**	6.0 (16.5)**
Viability (%)	69.5 (24.8)	78 (16)	86.5 (14.5)	72.5 (29.5)
Neutrophils, %	49.3 (36.1)	81.5 (28.5)*	91.0 (7.6)**	83.5 (25.3)*
Neutrophils count(x10^3^/g sputum)	120.2 (532)	4511.8 (5853)*	11139.0 (24009)**	3523.8 (16974)**
Macrophages, %	32.0 (26.1)	8 (14.7)*	4.5 (7.1)**	6.5 (10.9)**
Eosinophils, %	0.0 (0.6)	2.5 (3.8)*	0.4 (0.6)	0.5 (1)
Lymphocytes, %	1.9 (2.6)	1.0 (1.8)	1.0 (1.0)	1.3 (2.8)
Epithelial cells, %	6.8 (21.8)	7.0 (7.9)	1.8 (3.6)	1.1 (7.9)
**Exhaled breath condensate**				
LTB_4_ pg/mL	34.0 (28)	74.0 (53)*	66.0 (47.0)**	68 (46)*
GRO-α pg/mL	10.0 (13)	17 (34)	56.1 (49.0)*	45.0 (46.0)*

Data are expressed as medians (IQR), or mean cells counts are expressed as absolute counts per gramme (g) of sputum and as percentages of total inflammatory cells, excluding squamous cells. COPD: chronic obstructive pulmonary disease.

*p < 0.05, **p < 0.001, ***p <0.0001 vs healthy subjects, †p <0.05, †† p <0.01, ††† p <0.001 vs stable COPD.

**Table 3:** **Changes in functional parameters, blood cells and markers of inflammation, induced sputum cells, EBC LTB**_**4**_ **and GRO-α during an acute exacerbation phase and a stable phase of COPD**

	**Outpatients (n = 16)**		**Hospitalized patients (n = 11)**	
	Exacerbated phase	Stable phase	Exacerbated phase	Stable phase
**Functional parameters**				
FEV1, % predicted	48.1 ± 2.9	56.8 ± 3.4****	36.1 ± 4.6	42.7 ± 5.9*
FEV1/FVC %	47.0 ± 3.3	49.0 ± 3.5	47.9 ± 2.6	49.7 ± 2.6
**Blood**				
Leukocytes (per mL)	8825 (5040)	7045 (1862)*	12660 (5560)	8960 (4550)**
Neutrophils (per mL)	6670 (5093)	4545 (2668)*	9090 (5485)	5890 (3720)**
Neutrophils, %	76.0 (8.4)	64.4 (23.0)****	85.3 (14.8)	68.5 (11.0)**
Lymphocytes, %	17.0 (10.1)	23.6 (20.5)*	9.6 (8.5)	18.2 (5.7)*
CRP (mg/L)	21.4 (83.5)	1.8 (2.0)***	8.0 (52.4)	5.5 (6.0)*
Fibrinogen (g/L)	5.5 ± 0.5	3.4 ± 0.3***	4.7 ± 0.4	4.4 ± 0.4
**Induced sputum**				
Squamous epithelial cells (%)	0.0 (2.0)	6.5 (9.5)	10.0 (1.0)	23.0 (4.0)
Total non squamous cell count (x 10^6^ cells/g sputum)	13.1 (25.7)	7.2 (8.8)	5.4 (16.3)	3.7 (6.2)*
Neutrophils, %	91.0 (7.6)	81.1 (28.9)**	82.0 (24.6)	80.0 (19.5)
Neutrophils count(x10^3^/g sputum)	11139.0 (24009.0)	4688.0 (6655.0)*	2107.4 (15597.0)	2332.8 (4304.0)
Macrophages, %	4.5 (7.1)	7.8 (14.0)	10.0 (11)	8.5 (9)
Eosinophils, %	0.4 (0.6)	1.0 (1.7)	0.5 (3.9)	1.0 (9.8)
Lymphocytes, %	1.0 (1.0)	2.0 (5.2)	1.5 (2.5)	1.5 (1.9)
**Exhaled breath condensate**				
LTB4 pg/mL	66.0 (47)	47.0 (29.0)**	67.0 (36.0)	47.0 (30.0)
GRO alpha pg/mL	56.1 (49.0)	31.0 (29.0)**	45.0 (46.0)	15.1 (17.0)*

Functional data are expressed as means ± SD. Blood, sputum and EBC data are expressed as medians (IQR). *p < 0.05; ** p < 0.01, ***p < 0.005, **** p < 0.001.

### EBC Neutrophil chemotactic activity

EBC NCA from stable COPD, outpatients with AECOPD, hospitalized patients with AECOPD and healthy subjects displayed all significant neutrophil NCA (median (IQR) CI (chemotactic index): 2.3 (0.7) p < 0.0001; CI: 2.9 (1.5) p < 0.01, CI: 1.8 (1.8) p < 0.01 and CI 1.0 (1.4), p < 0.05 respectively). Overall NCA in EBC correlated with sputum neutrophil counts (r = 0.26, p < 0.05).

**Figure 5:**
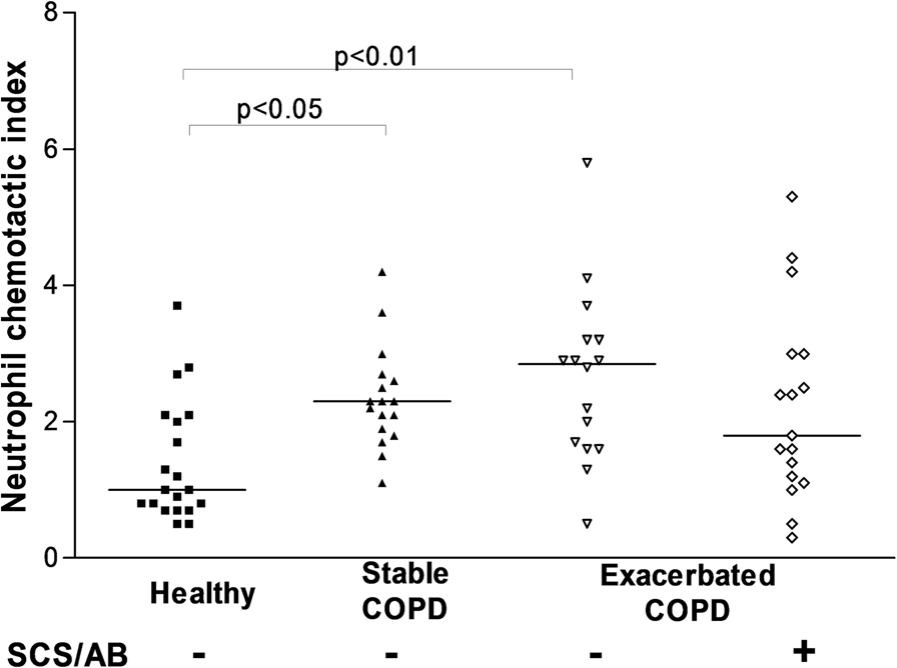
**EBC neutrophil chemotactic activity from stable and exacerbated COPD patients and healthy ex-smokers subjects.** Horizontal bars represent the median. SCS-AB: treatment with antibiotics and systemic corticosteroids before EBC collection.

Stable COPD and outpatients with AECOPD, but not hospitalized COPD patients, had raised EBC NCA compared to healthy subjects (p < 0.05 and p < 0.01 respectively) (Figure 5).

**Figure 6:**
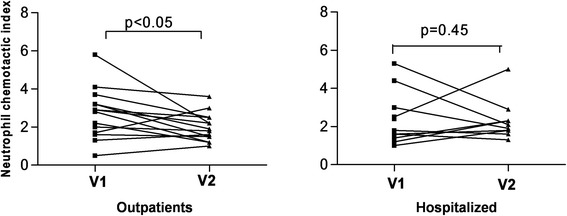
**EBC neutrophil chemotactic activity in acute phase (V1) and stable phase (V2) of outpatients and hospitalized patients with acute exacerbation of COPD.**

In outpatients with AECOPD there was a significant fall in EBC NCA after recovery (p < 0.05) (Figure 6), a phenomenon not observed in hospitalized AECOPD (p = 0.27).

In exacerbated outpatients and hospitalized COPD patients, there were no significant difference in EBC NCA between patients with or without a positive sputum bacterial culture (p = 0.16 and p = 0.14 respectively). Likewise there was no significant difference in EBC NCA between those with or without purulent sputum. Moreover, in outpatients with AECOPD, EBC NCA at day 1 and day 42 were not different between patients treated or not treated by systemic steroids for their AECOPD (p =0.92 and p =0.43 respectively).

### LTB_4_ and GRO-α values in exhaled breath condensate

We found a significant increase of LTB_4_ level in EBC from stable COPD and exacerbated patients by comparison to healthy controls (Table 2). The detection rate of EBC LTB_4_ was higher (p < 0.05) in outpatients and hospitalised patients with AECOPD (100% in both circumstance) by comparison with controls (70%), but not with stable COPD patients (94%). After recovery of the AECOPD, levels of EBC LTB_4_ significantly fell by comparison to the acute phase in outpatients (Table 3) and tended to do so for hospitalized patients. There was a fall in mean EBC LTB_4_ levels of 9 pg/mL (95% Confidence Interval:-12 - 30) and 35 pg/mL (95% Confidence Interval: 11 – 60) for hospitalized and outpatients with AECOPD respectively.

Compared to healthy subjects EBC GRO-α level was only increased during exacerbation of COPD (Table 2) whereas there was no difference between stable COPD and healthy controls. The detection rate of EBC GRO-α was higher (p < 0.001) in outpatients and hospitalised patients with AECOPD (75% and 82% respectively) by comparison with controls and stable COPD patients (30% and 53% respectively). At recovery, EBC GRO-α level significantly fell in both groups of AECOPD (Table 3). We found a fall in mean EBC GRO-α level of 29 pg/mL (95% Confidence Interval: 14–73) and 22 pg/mL (95% Confidence Interval: 7– 37) for hospitalized and outpatients with AECOPD respectively.

### Correlation of EBC chemokines with EBC NCA and sputum cells

For the whole population, there was a weak positive correlation between EBC LTB_4_ level and both sputum neutrophil counts (r = 0.43, p < 0.001) and EBC NCA (r = 0.34, p < 0.01). There was also a weak positive correlation between EBC GRO-α and EBC NCA (r = 0.31, p < 0.05).

### Determination of the contribution of GRO-α to EBC neutrophil chemotaxis

Increasing concentrations (0.1 to 10 μg/mL) of our anti-human GRO-α monoclonal antibody had no effect on EBC neutrophil chemotactic activity of exacerbated COPD patients (n = 10) (data not shown).

## Discussion

In this study, we have clearly confirmed that EBC from ex-smoking COPD patients contained raised neutrophil chemotactic activity as compared to healthy ex-smokers. The new finding is that the EBC neutrophil chemotactic activity further increased during exacerbation but only in those patients not already treated by antibiotics and systemic steroids at the time of EBC collection. The EBC neutrophil chemotactic activity for the whole population was weakly correlated to sputum neutrophil count and to EBC LTB_4_ and GRO-α levels. Furthermore, the latter two neutrophil chemotactic agents were found to be increased in EBC during AECOPD when compared to healthy controls, while significantly decreasing at recovery time.

EBC from ex-smoking healthy subjects and COPD patients contain active soluble chemoattractants for neutrophils as demonstrated by a significant neutrophil chemotactic activity. Previous studies have shown a neutrophil chemotactic activity in the EBC of healthy subjects [17,18,29] and COPD patients [17,18]. In addition cigarette smoking by itself was also found to increase neutrophil chemotactic activity [17,29]. Thus, to be independent of the tobacco influence, we decided in this study to work only with ex-smokers. As a result, we confirm that EBC from stable COPD patients display greater neutrophil chemotactic activity than those from healthy controls. In spite of some overlapping of the data between exacerbated and stable phase which prevent us to draw strong conclusions, we showed, for the first time, an increase in EBC neutrophil chemotactic activity during exacerbation of outpatients with COPD when compared to the recovery period. Our findings are in keeping with the fact that neutrophilic airways inflammation is indeed a major feature of COPD both in stable phase [1,4,30–32] and during exacerbation where neutrophils are even more attracted into the airways [6,8,33,34]. This obviously does not exclude that some COPD patients may have raised sputum eosinophilia but the proportion of these patients is less than 20% both in stable phase and during exacerbation in our study.

If the majority of the patients were clinically improved by treatment with antibiotics and/or steroids, it doesn’t mean that they had all an infectious exacerbation, keeping in mind that positive culture could be also the consequence of a bronchial colonization, and that a negative culture cannot exclude an infectious exacerbation due to viral infection. Our intention was to focus on exacerbation whichever its trigger. It was also shown that neutrophil inflammatory markers declined to pre-exacerbation levels after treatment [35–37]. It is well known that inflammation is amplified during exacerbations. Sputum markers of airway neutrophilic inflammation (myeloperoxidase, neutrophil elastase, IL-8, LTB_4_) were shown to increase during acute COPD exacerbation [21,35–38]. It was also shown that neutrophil inflammatory markers declined to pre-exacerbation levels after treatment [35–37].

Moreover, EBC markers of inflammation such as LTB_4_ [13,23] several cytokines including TNF-α, IL1-B,IL-6 and IL-8 [9,39], as well as markers of oxidative stress like isoprostane [13,23,40] and hydrogen peroxide [23,41,42] were also found to rise during exacerbation while diminishing following treatment or when returned to stable phase [13,14,23,41,42].

Indeed LTB_4_ is a potent chemoattractant of neutrophils, and it may be released by macrophages, epithelial cells and activated neutrophils [43]. Whereas EBC and sputum represent different airway compartment, LTB_4_ was found to be raised in EBC [13,18,44,45] or sputum [18,44] of stable COPD, and further increased during exacerbations while diminishing following antibiotic treatment [13,21,23]. Our study confirmed these results in stable and exacerbated COPD patients. However in hospitalized COPD patients treated by systemic corticosteroid and antibiotics for 3 days before EBC collection there was no significant fall at 6 weeks after treatment. The lack of difference between EBC LTB_4_ level and NCA during hospitalization and at 6 weeks is likely to be secondary to the anti-inflammatory effect of the treatment already given at hospital before EBC collection. This is in agreement with the study of Ko et al. [14] where changes in inflammatory mediators including LTB_4_ were not apparent during the course of AECOPD leading to hospitalization when day 5 and 60 were considered. The time point at which sample is collected during an exacerbation seems to be critical. Our results suggest that the increased neutrophil chemotactic activity during exacerbation might be partly due to elevated LTB4, although experiments with selective LTB4 receptor antagonists are required.

In our study, we demonstrated for the first time that GRO-α, a CXC chemokine with strong activating and chemotactic abilities for neutrophils, was found to be raised in the EBC from exacerbated, but not stable, COPD patients. It would suggest a role of this chemokine in the recruitment and activation of neutrophils during an AECOPD. A previous study showed an increased of GRO-α in sputum, but not in bronchoalveolar lavage fluid, of stable smoking COPD patients by comparison with smoking and non smoking healthy controls [46]. However, it is admitted that EBC and sputum reflect different airway compartments. Moreover, a recent study has demonstrated that GRO-α could be detected in EBC of COPD and healthy subjects, and that EBC GRO-α level is lower in stable ex-smoking COPD by comparison with controls [47]. In the same study, the authors did not find any difference of EBC GRO-α level between COPD patients with and without inhaled corticosteroid therapy [47]. They, however, did not measure how GRO-a level varied during an exacerbation. Despite our observation that GRO-α could be a mediator essentially involved in the rise of neutrophilic inflammation during an exacerbation, we didn’t find any effect of our anti-human GRO-α monoclonal antibody on EBC neutrophil chemotactic activity of exacerbated COPD patients. We could explain this discrepancy by instability of neutrophil membranair expression of CXCR2 (ligands are GRO-α and IL8) and CXCR1 (ligand is IL8) [48], and that there is evidence for a functional distinction between polymorphonuclear neutrophil CXCR1 and CXCR2 in inflammatory conditions [49]. In addition, EBC, by contrast to a pure solution of GRO-α, is a soap of chemoattractants some of which like IL-8 may actually modulate CXCR1/2 expression [47] and thereby the neutrophil response to a chemoattractant. Of course we cannot rule out the possibility that GRO-α, though being a potent agent in a simple *in vitro* model, might only marginally contribute to the overall chemotactic activity contained in a complex mixture like EBC. Beside EBC there are today new omics techniques available [50] including nuclear magnetic resonance spectroscopy of EBC [51–53] and electronic noses [54] that can be used for more complete assessment of respiratory inflammation in stable and exacerbated COPD.

## Conclusion

This study has shown that EBC from ex-smoking COPD contained raised neutrophil chemotactic activity as compared to healthy ex-smokers, with a further amplification during exacerbation of patient not already treated by antibiotic and systemic steroids. Furthermore LTB_4_ and GRO-α, two neutrophil chemotactic agents, were found to be increased in EBC during AECOPD while significantly decreased at recovery time.

## Abbreviations

AB: Antibiotics
AECOPD: Acute Exacerbation of Chronic Obstructive Lung Disease
BSA: Bovine serum albumine
CI: Chemotactic index
COPD: Chronic obstructive pulmonary disease
CRP: C-reactive protein
EBC: Exhaled breath condensate
ES: Exsmokers
FEV_1_: Forced expiratory volume in one second
FVC: Forced volume capacity
GOLD: Global Initiative for Chronic Obstructive Lung Disease
GROα: Growth related oncogene-alpha
HBSS: Hank’s balanced salt solution
HES: Healthy exsmokers
ICS: Inhaled corticosteroid
IQR: Interquartile range
IS: Induced sputum
LTB_4_: Leukotriene B4
NCA: Neutrophil chemotactic activity
SCS: Systemic corticosteroid
SD: Standard deviation
SEM: Standard error of the mean

## References

[1] Stockley RA (2002). Neutrophils and the pathogenesis of COPD. Chest.

[2] Hogg JC, Chu F, Utokaparch S, Woods R, Elliott WM, Buzatu L, Cherniack RM, Rogers RM, Sciurba FC, Coxson HO, Pare PD (2004). The nature of small-airway obstruction in chronic obstructive pulmonary disease. N Engl J Med.

[3] O’Donnell RA, Peebles C, Ward JA, Daraker A, Angco G, Broberg P, Pierrou S, Lund J, Holgate ST, Davies DE, Delany DJ, Wilson SJ, Djukanovic R (2004). Relationship between peripheral airway dysfunction, airway obstruction, and neutrophilic inflammation in COPD. Thorax.

[4] Peleman RA, Rytila PH, Kips JC, Joos GF, Pauwels RA (1999). The cellular composition of induced sputum in chronic obstructive pulmonary disease. Eur Respir J.

[5] Marin A, Monso E, Garcia-Nunez M, Sauleda J, Noguera A, Pons J, Agusti A, Morera J (2010). Variability and effects of bronchial colonisation in patients with moderate COPD. Eur Respir J.

[6] Stanescu D, Sanna A, Veriter C, Kostianev S, Calcagni PG, Fabbri LM, Maestrelli P (1996). Airways obstruction, chronic expectoration, and rapid decline of FEV1 in smokers are associated with increased levels of sputum neutrophils. Thorax.

[7] Papi A, Bellettato CM, Braccioni F, Romagnoli M, Casolari P, Caramori G, Fabbri LM, Johnston SL (2006). Infections and airway inflammation in chronic obstructive pulmonary disease severe exacerbations. Am J Respir Crit Care Med.

[8] Saetta M, Di SA, Maestrelli P, Turato G, Ruggieri MP, Roggeri A, Calcagni P, Mapp CE, Ciaccia A, Fabbri LM (1994). Airway eosinophilia in chronic bronchitis during exacerbations. Am J Respir Crit Care Med.

[9] Horvath I, Hunt J, Barnes PJ, Alving K, Antczak A, Baraldi E, Becher G, van Beurden WJ, Corradi M, Dekhuijzen R, Dweik RA, Dwyer T, Effros R, Erzurum S, Gaston B, Gessner C, Greening A, Ho LP, Hohlfeld J, Jobsis Q, Laskowski D, Loukides S, Marlin D, Montuschi P, Olin AC, Redington AE, Reinhold P, van Rensen EL, Rubinstein I, Silkoff P (2005). Exhaled breath condensate: methodological recommendations and unresolved questions. Eur Respir J.

[10] Montuschi P (2005). Exhaled breath condensate analysis in patients with COPD. Clin Chim Acta.

[11] Montuschi P, Santini G, Valente S, Mondino C, Macagno F, Cattani P, Zini G, Mores N (2014). Liquid chromatography-mass spectrometry measurement of leukotrienes in asthma and other respiratory diseases. J Chromatogr B Analyt Technol Biomed Life Sci.

[12] Corhay JL, Vincken W, Schlesser M, Bossuyt P, Imschoot J (2013). Chronic bronchitis in COPD patients is associated with increased risk of exacerbations: a cross-sectional multicentre study. Int J Clin Pract.

[13] Biernacki WA, Kharitonov SA, Barnes PJ (2003). Increased leukotriene B4 and 8-isoprostane in exhaled breath condensate of patients with exacerbations of COPD. Thorax.

[14] Ko FW, Leung TF, Wong GW, Ngai J, To KW, Ng S, Hui DS (2009). Measurement of tumor necrosis factor-alpha, leukotriene B4, and interleukin 8 in the exhaled breath condensate in patients with acute exacerbations of chronic obstructive pulmonary disease. Int J Chron Obstruct Pulmon Dis.

[15] Cunningham S, McColm JR, Ho LP, Greening AP, Marshall TG (2000). Measurement of inflammatory markers in the breath condensate of children with cystic fibrosis. Eur Respir J.

[16] Simpson JL, Wood LG, Gibson PG (2005). Inflammatory mediators in exhaled breath, induced sputum and saliva. Clin Exp Allergy.

[17] Corhay JL, Hemelaers L, Henket M, Sele J, Louis R (2007). Granulocyte chemotactic activity in exhaled breath condensate of healthy subjects and patients with COPD. Chest.

[18] Corhay JL, Henket M, Nguyen D, Duysinx B, Sele J, Louis R (2009). Leukotriene B4 contributes to exhaled breath condensate and sputum neutrophil chemotaxis in COPD. Chest.

[19] Vestbo J, Hurd SS, Agusti AG, Jones PW, Vogelmeier C, Anzueto A, Barnes PJ, Fabbri LM, Martinez FJ, Nishimura M, Stockley RA, Sin DD, Rodriguez-Roisin R (2013). Global strategy for the diagnosis, management, and prevention of chronic obstructive pulmonary disease: GOLD executive summary. Am J Respir Crit Care Med.

[20] Bhowmik A, Seemungal TA, Sapsford RJ, Wedzicha JA (2000). Relation of sputum inflammatory markers to symptoms and lung function changes in COPD exacerbations. Thorax.

[21] Crooks SW, Bayley DL, Hill SL, Stockley RA (2000). Bronchial inflammation in acute bacterial exacerbations of chronic bronchitis: the role of leukotriene B4. Eur Respir J.

[22] Hill AT, Campbell EJ, Hill SL, Bayley DL, Stockley RA (2000). Association between airway bacterial load and markers of airway inflammation in patients with stable chronic bronchitis. Am J Med.

[23] Antczak A, Ciebiada M, Pietras T, Piotrowski WJ, Kurmanowska Z, Gorski P (2012). Exhaled eicosanoids and biomarkers of oxidative stress in exacerbation of chronic obstructive pulmonary disease. Arch Med Sci.

[24] Celli BR, MacNee W (2004). Standards for the diagnosis and treatment of patients with COPD: a summary of the ATS/ERS position paper. Eur Respir J.

[25] Rodriguez-Roisin R (2000). Toward a consensus definition for COPD exacerbations. Chest.

[26] Stockley RA, O’Brien C, Pye A, Hill SL (2000). Relationship of sputum color to nature and outpatient management of acute exacerbations of COPD. Chest.

[27] Anthonisen NR, Manfreda J, Warren CP, Hershfield ES, Harding GK, Nelson NA (1987). Antibiotic therapy in exacerbations of chronic obstructive pulmonary disease. Ann Intern Med.

[28] Delvaux M, Henket M, Lau L, Kange P, Bartsch P, Djukanovic R, Louis R (2004). Nebulised salbutamol administered during sputum induction improves bronchoprotection in patients with asthma. Thorax.

[29] Garey KW, Neuhauser MM, Robbins RA, Danziger LH, Rubinstein I (2004). Markers of inflammation in exhaled breath condensate of young healthy smokers. Chest.

[30] Ronchi MC, Piragino C, Rosi E, Amendola M, Duranti R, Scano G (1996). Role of sputum differential cell count in detecting airway inflammation in patients with chronic bronchial asthma or COPD. Thorax.

[31] Thurlbeck WM (1990). Pathology of chronic airflow obstruction. Chest.

[32] Malerba M, Montuschi P (2012). Non-invasive biomarkers of lung inflammation in smoking subjects. Curr Med Chem.

[33] Qiu Y, Zhu J, Bandi V, Atmar RL, Hattotuwa K, Guntupalli KK, Jeffery PK (2003). Biopsy neutrophilia, neutrophil chemokine and receptor gene expression in severe exacerbations of chronic obstructive pulmonary disease. Am J Respir Crit Care Med.

[34] Ras G, Wilson R, Todd H, Taylor G, Cole P (1990). Effect of bacterial products on neutrophil migration in vitro. Thorax.

[35] Aaron SD, Angel JB, Lunau M, Wright K, Fex C, Le SN, Dales RE (2001). Granulocyte inflammatory markers and airway infection during acute exacerbation of chronic obstructive pulmonary disease. Am J Respir Crit Care Med.

[36] Gompertz S, O’Brien C, Bayley DL, Hill SL, Stockley RA (2001). Changes in bronchial inflammation during acute exacerbations of chronic bronchitis. Eur Respir J.

[37] White AJ, Gompertz S, Bayley DL, Hill SL, O’Brien C, Unsal I, Stockley RA (2003). Resolution of bronchial inflammation is related to bacterial eradication following treatment of exacerbations of chronic bronchitis. Thorax.

[38] Fujimoto K, Yasuo M, Urushibata K, Hanaoka M, Koizumi T, Kubo K (2005). Airway inflammation during stable and acutely exacerbated chronic obstructive pulmonary disease. Eur Respir J.

[39] Gessner C, Scheibe R, Wotzel M, Hammerschmidt S, Kuhn H, Engelmann L, Hoheisel G, Gillissen A, Sack U, Wirtz H (2005). Exhaled breath condensate cytokine patterns in chronic obstructive pulmonary disease. Respir Med.

[40] Montuschi P, Barnes PJ, Ciabattoni G (2010). Measurement of 8-isoprostane in exhaled breath condensate. Methods Mol Biol.

[41] Gerritsen WB, Asin J, Zanen P, van den Bosch JM, Haas FJ (2005). Markers of inflammation and oxidative stress in exacerbated chronic obstructive pulmonary disease patients. Respir Med.

[42] Oudijk EJ, Gerritsen WB, Nijhuis EH, Kanters D, Maesen BL, Lammers JW, Koenderman L (2006). Expression of priming-associated cellular markers on neutrophils during an exacerbation of COPD. Respir Med.

[43] Ford-Hutchinson AW, Bray MA, Doig MV, Shipley ME, Smith MJ (1980). Leukotriene B, a potent chemokinetic and aggregating substance released from polymorphonuclear leukocytes. Nature.

[44] Kostikas K, Gaga M, Papatheodorou G, Karamanis T, Orphanidou D, Loukides S (2005). Leukotriene B4 in exhaled breath condensate and sputum supernatant in patients with COPD and asthma. Chest.

[45] Montuschi P, Kharitonov SA, Ciabattoni G, Barnes PJ (2003). Exhaled leukotrienes and prostaglandins in COPD. Thorax.

[46] Traves SL, Culpitt SV, Russell RE, Barnes PJ, Donnelly LE (2002). Increased levels of the chemokines GROalpha and MCP-1 in sputum samples from patients with COPD. Thorax.

[47] Ko FW, Lau CY, Leung TF, Wong GW, Lam CW, Hui DS (2006). Exhaled breath condensate levels of 8-isoprostane, growth related oncogene alpha and monocyte chemoattractant protein-1 in patients with chronic obstructive pulmonary disease. Respir Med.

[48] Hu N, Westra J, Rutgers A, Doornbos-Van der Meer B, Huitema MG, Stegeman CA, Abdulahad WH, Satchell SC, Mathieson PW, Heeringa P, Kallenberg CG (2011). Decreased CXCR1 and CXCR2 expression on neutrophils in anti-neutrophil cytoplasmic autoantibody-associated vasculitides potentially increases neutrophil adhesion and impairs migration. Arthritis Res Ther.

[49] Stillie R, Farooq SM, Gordon JR, Stadnyk AW (2009). The functional significance behind expressing two IL-8 receptor types on PMN. J Leukoc Biol.

[50] Wheelock CE, Goss VM, Balgoma D, Nicholas B, Brandsma J, Skipp PJ, Snowden S, Burg D, D’Amico A, Horvath I, Chaiboonchoe A, Ahmed H, Ballereau S, Rossios C, Chung KF, Montuschi P, Fowler SJ, Adcock IM, Postle AD, Dahlen SE, Rowe A, Sterk PJ, Auffray C, Djukanovic R (2013). Application of ‘omics technologies to biomarker discovery in inflammatory lung diseases. Eur Respir J.

[51] de LG, Paris D, Melck D, Montuschi P, Maniscalco M, Bianco A, Sofia M, Motta A (2013). Separating smoking-related diseases using NMR-based metabolomics of exhaled breath condensate. J.Proteome.Res.

[52] Montuschi P, Paris D, Melck D, Lucidi V, Ciabattoni G, Raia V, Calabrese C, Bush A, Barnes PJ, Motta A (2012). NMR spectroscopy metabolomic profiling of exhaled breath condensate in patients with stable and unstable cystic fibrosis. Thorax.

[53] Motta A, Paris D, Melck D, De LG, Maniscalco M, Sofia M, Montuschi P (2012). Nuclear magnetic resonance-based metabolomics of exhaled breath condensate: methodological aspects. Eur Respir J.

[54] Montuschi P, Mores N, Trove A, Mondino C, Barnes PJ (2013). The electronic nose in respiratory medicine. Respiration.

